# ProSAAS-Derived Peptides are Colocalized with Neuropeptide Y and Function as Neuropeptides in the Regulation of Food Intake

**DOI:** 10.1371/journal.pone.0028152

**Published:** 2011-12-02

**Authors:** Jonathan H. Wardman, Iryna Berezniuk, Shi Di, Jeffrey G. Tasker, Lloyd D. Fricker

**Affiliations:** 1 Department of Molecular Pharmacology, Albert Einstein College of Medicine, New York, New York, United States of America; 2 Dominick P. Purpura Department of Neuroscience, Albert Einstein College of Medicine, New York, New York, United States of America; 3 Department of Cell and Molecular Biology, Tulane University, New Orleans, Louisiana, United States of America; 4 Neuroscience Program, Tulane University, New Orleans, Louisiana, United States of America; Sapienza University of Rome, Italy

## Abstract

ProSAAS is the precursor of a number of peptides that have been proposed to function as neuropeptides. Because proSAAS mRNA is highly expressed in the arcuate nucleus of the hypothalamus, we examined the cellular localization of several proSAAS-derived peptides in the mouse hypothalamus and found that they generally colocalized with neuropeptide Y (NPY), but not α-melanocyte stimulating hormone. However, unlike proNPY mRNA, which is upregulated by food deprivation in the mediobasal hypothalamus, neither proSAAS mRNA nor proSAAS-derived peptides were significantly altered by 1–2 days of food deprivation in wild-type mice. Furthermore, while proSAAS mRNA levels in the mediobasal hypothalamus were significantly lower in *Cpe^fat/fat^* mice as compared to wild-type littermates, proNPY mRNA levels in the mediobasal hypothalamus and in other subregions of the hypothalamus were not significantly different between wild-type and *Cpe^fat/fat^* mice. Intracerebroventricular injections of antibodies to two proSAAS-derived peptides (big LEN and PEN) significantly reduced food intake in fasted mice, while injections of antibodies to two other proSAAS-derived peptides (little LEN and little SAAS) did not. Whole-cell patch clamp recordings of parvocellular neurons in the hypothalamic paraventricular nucleus, a target of arcuate NPY projections, showed that big LEN produced a rapid and reversible inhibition of synaptic glutamate release that was spike independent and abolished by blocking postsynaptic G protein activity, suggesting the involvement of a postsynaptic G protein-coupled receptor and the release of a retrograde synaptic messenger. Taken together with previous studies, these findings support a role for proSAAS-derived peptides such as big LEN as neuropeptides regulating food intake.

## Introduction

**Figure 1:**
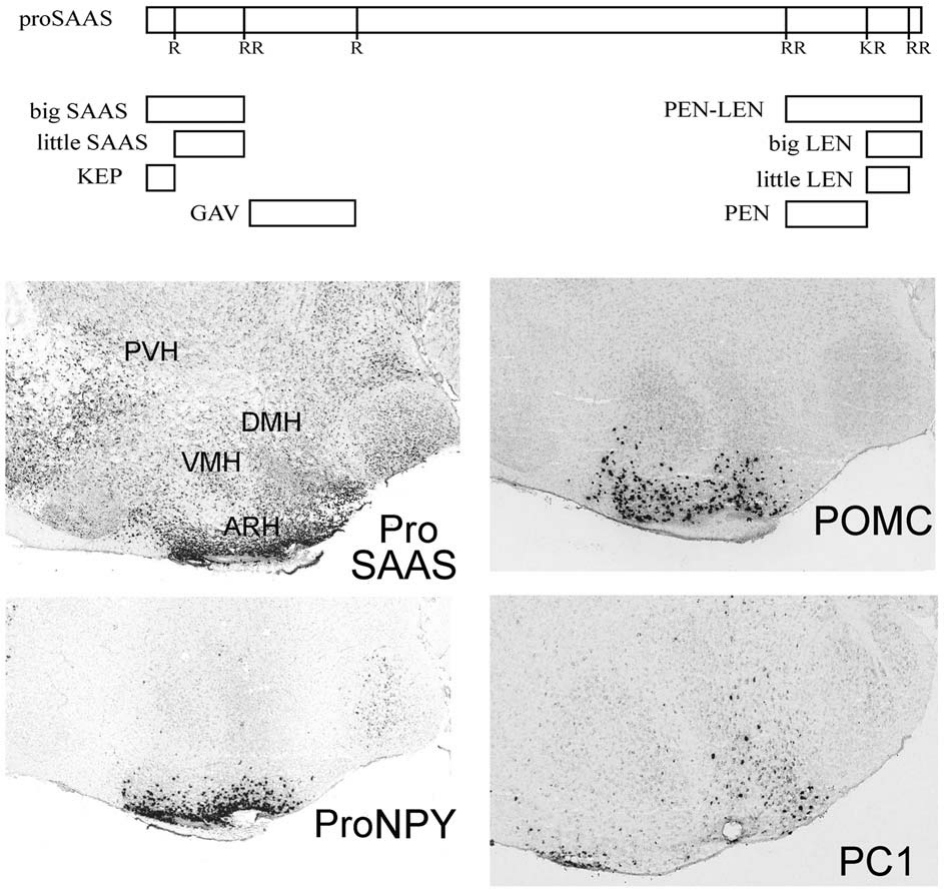
Schematic of proSAAS and proSAAS-derived peptides, and the localization of proSAAS, proNPY, POMC, and PC1/3 mRNA in the hypothalamus of the mouse. Top panel: Schematic diagram showing the major proSAAS-derived peptides previously detected in various brain regions and other relevant peptides. The relative size and position of these peptides within proSAAS are indicated. Bottom panels: Images of proSAAS, proNPY, POMC, and PC1/3 mRNA distribution were downloaded from the Allen Mouse Brain Atlas [http://mouse.brain-map.org], Seattle, WA, Allen Institute for Brain Science ©2009 [50].

Peptides play major roles in diverse physiological functions, including as hormones, neurotransmitters, and growth factors [1], [2]. Bioactive peptides are typically produced by cleavage of larger precursor proteins at specific sites containing basic residues, often in pairs such as Arg-Arg or Lys-Arg [3], [4]. Endopeptidases such as prohormone convertase (PC) 1/3 and 2 cut the precursors at these sites, generating intermediate peptides with C-terminal basic amino acid extensions [5], [6]. Carboxypeptidases then trim these basic residues from the ends of the peptides, after which the peptides may need further modification (such as amidation) before the peptides are biologically active [7]. Carboxypeptidase E (CPE) is the major peptide-processing carboxypeptidase; this enzyme is found in all neuroendocrine tissues and cleaves many C-terminally extended peptides to generate the mature bioactive form [8]. The *fat* mutation, a spontaneous mouse mutation, was found to be a point mutation in CPE that causes the peptidase to be inactive [9]. Mice homozygous for this mutation, which was renamed *Cpe^fat^*, show an accumulation of the peptide processing intermediates containing basic residues on their C-termini and a reduction in levels of fully processed peptides [10]. An affinity chromatography technique was used to isolate these neuropeptide intermediates after which the peptides were sequenced using tandem mass spectrometry [11]. This approach identified the processing intermediates of many previously characterized neuroendocrine peptides as well as several novel peptides. Five of these novel peptides were named based on primary amino acid sequences contained within the larger peptides: big SAAS, little SAAS, PEN, big LEN, and little LEN. All five of these peptides were derived from the same precursor, named proSAAS, by cleavage at basic amino acid-containing sites (Figure 1). The involvement of CPE in the biosynthesis of proSAAS-derived peptides indicates that these peptides are processed in the regulated secretory pathway of neuroendocrine cells.

Although there is no sequence similarity among proSAAS and other proteins, there are some features in common between proSAAS and 7B2, a member of the granin family [12]. Both proteins are broadly expressed in brain and neuroendocrine tissues [13], are generally the same size, and contain multiple cleavage sites for the PCs and CPE [11], [12], [14]. 7B2 is an inhibitor of PC2 [15], [16]. Similar studies with proSAAS showed that a small region at the boundary of PEN and LEN is a potent and selective inhibitor of PC1/3, but neither PEN nor LEN were able to inhibit PC1/3 [17]. Therefore, PEN, LEN, and peptides cleaved from the N-terminal region of proSAAS may serve other functions. ProSAAS is present in neuroendocrine cells lacking PC1/3, further suggesting that proSAAS-derived peptides may have functions independent of the role of proSAAS as a PC1/3 inhibitor [14].

Levels of proSAAS mRNA are highest in brain regions involved in feeding and body weight regulation, such as the arcuate nucleus and other regions of the hypothalamus [11]. Semi-quantitative *in situ* hybridization analysis of proSAAS mRNA in the Allen Mouse Brain Atlas shows highest levels in the arcuate nucleus (Figure S1). The pattern of proSAAS mRNA distribution in the medial hypothalamus is very similar to that of mRNAs encoding peptides known to function in body weight regulation, such as neuropeptide Y (NPY) and α-melanocyte stimulating hormone (α-MSH); the pattern of proSAAS mRNA distribution is distinct from that of PC1/3 mRNA (Figure 1). Transgenic mice overexpressing proSAAS showed a significant, late-onset gain in bodyweight, yet processing of peptides by PC1/3 appears normal in these mice, suggesting that the effect on bodyweight is not due to the inhibitory action of proSAAS and consistent with the possibility that the fully processed proSAAS-derived peptides have an orexigenic effect [18]. Furthermore, male mice lacking proSAAS show a 10–15% decrease in bodyweight, further suggesting that proSAAS-derived peptides have orexigenic effects [19]. Additional evidence that proSAAS-derived peptides function in body weight regulation was the finding that *Cpe^fat/fat^* mice have elevated levels of big LEN, relative to wild-type littermates [20]. Also, when *Cpe^fat/fat^* mice were deprived of food for 48 hours, the levels of several proSAAS-derived peptides increased several fold [21]. Taken together, these studies suggest a role for proSAAS-derived peptides in the control of body weight.

In the present study, we first examined if proSAAS-derived peptides colocalize with α-MSH or NPY in the arcuate nucleus using fluorescent double labeling immunohistochemistry. NPY is a well characterized orexigenic neuropeptide, whereas α-MSH is an established anorexigenic neuropeptide, both of which are expressed in distinct neurons of the hypothalamus. We also examined if proSAAS-derived peptides colocalized with green fluorescent protein (GFP) expressed under an NPY neuronal promoter. Because previous studies showed that proSAAS peptides were up-regulated in *Cpe^fat/fat^* mice, relative to wild-type mice, and further elevated in *Cpe^fat/fat^* mice subjected to food deprivation, we examined if levels of proSAAS mRNA and/or peptides were altered under these conditions. We also examined if intracerebroventricular (icv) injection of proSAAS-derived peptides or antibodies to these peptides affected food intake of mice. Previous studies established the orexigenic role of NPY by showing that direct injection of the peptide into brain stimulated feeding, whereas injection of anti-NPY antibodies reduced food intake [22], [23], [24]. A direct role for big LEN as a neuropeptide was further explored using whole-cell patch clamp recording of neurons located in the medial parvocellular region of the paraventricular nucleus. Because the paraventricular parvocellular neurons receive input from NPY-expressing neurons [25], these neurons should also be exposed to big LEN *in vivo* based on the general colocalization of big LEN and NPY. Taken together with previous studies, our results suggest that big LEN is a neuropeptide that functions in the control of food intake.

## Results

### Colocalization of proSAAS-derived peptides with NPY

**Figure 2:**
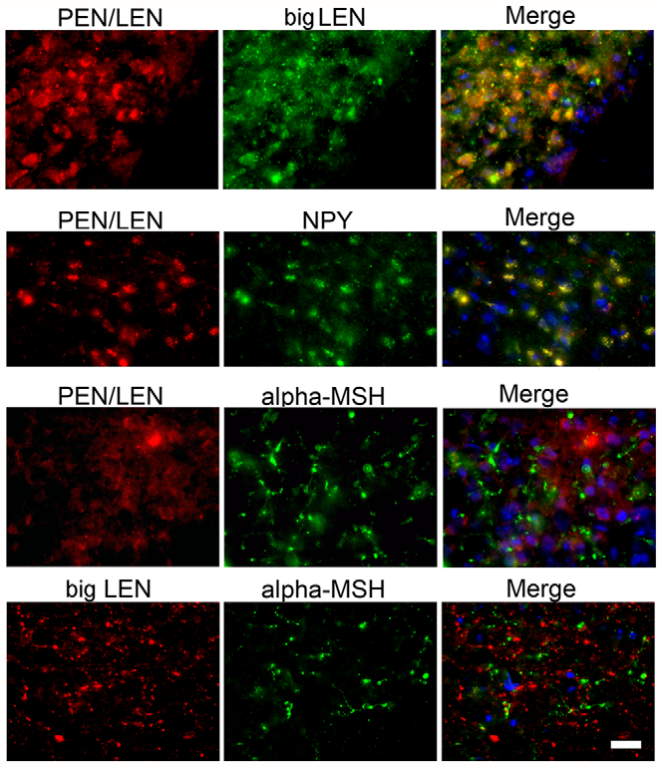
Coronal sections through mouse hypothalamus showing immunofluorescence colocalization of peptides in the arcuate nucleus. The upper panels show immunoreactive PEN/LEN peptides in red and immunoreactive big LEN peptides in green. The merged images (right) show general colocalization of the two peptides. The panels in the second row show immunoreactive PEN/LEN peptides in red and immunoreactive NPY peptides in green, and merged images show essentially complete colocalization of the two peptides. The panels in the third row show immunoreactive PEN/LEN peptides in red and immunoreactive α-MSH peptides in green. Merged images show no colocalization of the two peptides. The bottom row shows immunoreactive big LEN peptides in red and immunoreactive α-MSH peptides in green. Merged images show no colocalization of the two peptides. Nuclei are stained blue using DAPI (4,6- diamidino-2-phenylindole). The scale bar indicates 10 µm.

The Allen Mouse Brain Atlas shows the highest levels of proSAAS mRNA in the hypothalamus, specifically concentrated in the area of the arcuate nucleus (Figure 1 and Figure S1). This distribution pattern is similar to the distribution of mRNAs encoding NPY and proopiomelanocortin (POMC), the precursor of α-MSH (Figure 1). To test whether proSAAS-derived peptides colocalized with either NPY or α-MSH at the cellular level, we performed double immunofluorescence analyses with antisera to NPY, α-MSH, PEN-LEN, PEN and LEN. A chicken antiserum to the proSAAS peptide PEN-LEN and rabbit antisera to the peptides PEN and LEN were used together with commercially available antisera to NPY (rabbit) and α-MSH (sheep). PEN-LEN immunoreactivity was found throughout the mouse brain, with highest levels in the arcuate nucleus. A punctate staining pattern for PEN-LEN in the hypothalamic cell bodies suggested vesicular localization of these peptides (Figure 2). Co-staining of these same brain sections with an antibody against NPY showed strong immunoreactivity in the hypothalamus, also concentrated in the arcuate nucleus. When merged, images of the arcuate nucleus immunostained for both PEN-LEN and NPY showed considerable colocalization of these two peptides (Figure 2). These results indicate that some cells in the hypothalamus produce both proSAAS-derived peptides and NPY. In contrast, no overlap was observed when the arcuate nucleus was co-stained with α-MSH and either PEN-LEN or big LEN (Figure 2). The chicken anti-PEN-LEN antiserum showed extensive overlap with rabbit antisera to both big LEN (Figure 2) and PEN (not shown), as expected. The LEN antiserum used in these experiments was previously shown to be specific for big LEN and not recognize little LEN peptide, which lacks six C-terminal amino acids [26]. To confirm this, we performed blocking experiments and found that when the LEN antiserum was preincubated with big LEN peptide, there was a substantial decrease in immunofluorescence whereas similar experiments with little LEN peptide did not reduce the signal (Figure S2). Additional controls with preimmune serum showed negligible signals (Figure S2).

### ProSAAS- derived peptides in NPY peptidergic neurons

**Figure 3:**
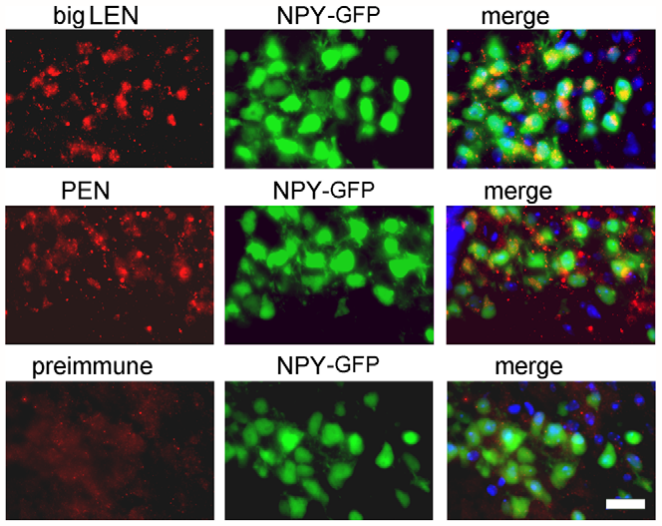
Coronal sections through the hypothalamus of NPY-GFP mice showing immunofluorescence localization of peptides in the arcuate nucleus. The upper panel shows immunoreactive big LEN peptides in red and NPY neurons expressing GFP in the cytosol in green. In the merged images big LEN peptide immunoreactivity can be seen in the green GFP-expressing neurons. The middle row of panels shows immunoreactive PEN peptides in red and NPY neurons in green. In the merged images, PEN peptide immunoreactivity can be seen in the green GFP-expressing neurons. The bottom panels show a section incubated with pre-immune serum and secondary Cy3 antibodies, with background staining only. Nuclei are stained blue using DAPI (4,6- diamidino-2-phenylindole). Scale bar 10 µm.

To directly test if immunoreactive PEN and big LEN colocalize with NPY-expressing neurons, we examined brain sections from mice expressing green fluorescent protein (GFP) under control of the NPY neuronal promoter. These mice show strong GFP fluorescence in the cell bodies of NPY neuropeptidergic neurons in the arcuate nucleus of the hypothalamus [27]. Many of these GFP-positive cells were positive for the expression of big LEN and PEN (Figure 3). Both big LEN and PEN immunoreactivity appeared with a punctate distribution, consistent with localization to secretory vesicles, while GFP under the control of the NPY promoter was expressed in the cytosol. Only low levels of background staining were observed when using preimmune serum (Figure 3).

### Quantitative real-time PCR (qRTPCR) in subhypothalamic brain regions

The finding that proSAAS-derived peptides and NPY are colocalized in many neurons raised the question of whether proSAAS and NPY are co-regulated. Previously, mRNA encoding NPY has been shown to be up-regulated by food deprivation [28]. Furthermore, in *Cpe^fat/fat^* mice, food deprivation was found to increase several proSAAS-derived peptide processing intermediates [21]. In addition, the C-terminal proSAAS peptide big LEN was found to be several-fold more abundant in *Cpe^fat/fat^* mouse hypothalamus compared to wild-type mouse hypothalamus [20]. Thus, we developed probes to detect proSAAS mRNA using qRTPCR so that analysis of mRNA levels could be performed in subregions of the hypothalamus. Probes for qRTPCR analysis of mouse proNPY mRNA were used to compare with the proSAAS mRNA.

**Figure 4:**
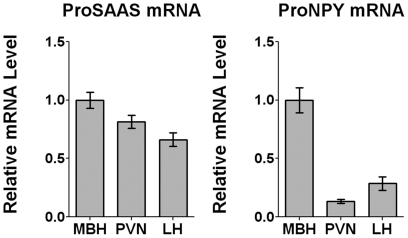
Levels of proSAAS mRNA and proNPY mRNA in subhypothalamic brain regions. Left: ProSAAS mRNA in the MBH, PVN, and LH in wild-type mice. Right: The MBH expresses the highest levels of proNPY mRNA, while the PVN and LH express much lower levels. Error bars show standard error of the mean (n = 8).

Relative levels of proSAAS and proNPY mRNA were quantified in the paraventricular nucleus (PVN), the lateral hypothalamus (LH), and the mediobasal hypothalamus (MBH) which includes the arcuate nucleus. The level of proSAAS mRNA was highest in the MBH, and slightly lower in the PVN and LH (Figure 4). These results generally match the distribution of proSAAS mRNA by *in situ* hybridization (Figure 1), considering that the MBH contains other regions in addition to the arcuate nucleus. ProNPY mRNA was much higher in the MBH than the other brain regions examined (Figure 4), consistent with the *in situ* hybridization results for this mRNA (Figure 1).

**Figure 5:**
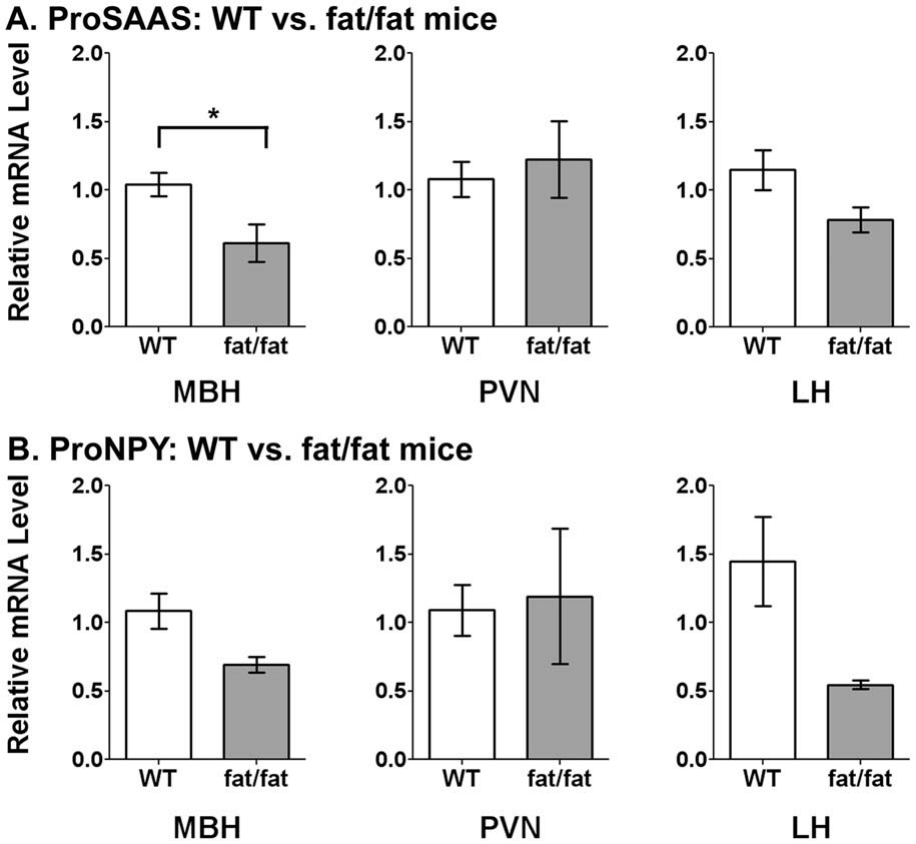
Comparison of relative levels of proSAAS mRNA and proNPY mRNA in wild-type versus *Cpe^fat/fat^* mice. A: proSAAS mRNA levels are significantly lower (p = 0.041) in the MBH of the *Cpe^fat/fat^* mice, relative to wild-type (WT) mice, and show a tendency to decrease in the LH of the *Cpe^fat/fat^* mice, but in this brain region the difference is not statistically significant (p = 0.23). B: proNPY mRNA levels show a tendency to decrease in the MBH and LH of *Cpe^fat/fat^* mice, relative to wild-type mice, but these changes are not statistically significant (p = 0.17 and 0.19, respectively). Error bars show standard error of the mean for n = 8. Abbreviations: WT, wild-type; fat/fat, *Cpe^fat/fat^*. *, p<0.05 using Student's two-tailed t-test.

To test if the previously observed elevated levels of big LEN in *Cpe^fat/fat^* mouse hypothalamus, relative to wild-type mouse hypothalamus [20], was due to increased proSAAS mRNA levels, qRTPCR analysis was used to compare *Cpe^fat/fat^* and wild-type mice. Surprisingly, levels of proSAAS mRNA decreased in the MBH and LH of *Cpe^fat/fat^* mice, relative to wild-type mice, although this difference was only statistically significant in the MBH (Figure 5A). Therefore, the 2-fold increase in big LEN previously observed in the *Cpe^fat/fat^* mouse hypothalamus is not due to an increase in proSAAS mRNA levels. ProNPY mRNA was also compared between *Cpe^fat/fat^* and wild-type mice and found to show a tendency to decrease in the MBH of *Cpe^fat/fat^* mice, although this change was not statistically significant (Figure 5B). In addition, proNPY mRNA showed a tendency to decrease in the LH of *Cpe^fat/fat^* mice, but this change was not statistically significant (Figure 5B).

**Figure 6:**
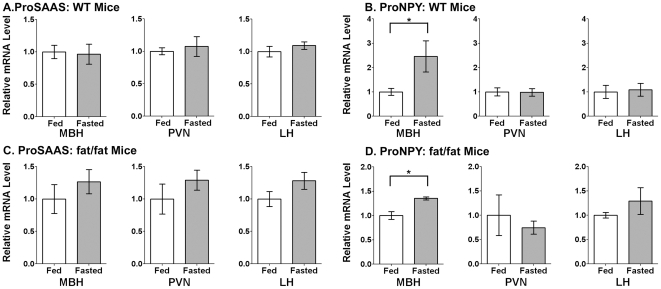
Comparison of proSAAS mRNA and proNPY mRNA levels in fed and fasted mice in various subhypothalamic brain regions. A. ProSAAS mRNA levels do not show a significant change in fasted WT mice relative to mice fed *ad libitum* in any of the brain regions examined. B. ProNPY mRNA levels increase significantly in the MBH of fasted mice relative to fed mice, but are not affected in the other two brain regions examined. C. ProSAAS mRNA levels do not show a significant change with fasting in subhypothalamic brain regions of *Cpe^fat/fat^* mice. D. ProNPY mRNA is significantly increased in the MBH of fasted *Cpe^fat/fat^* mice. Error bars show standard error of the mean for n = 8 in WT mice and n = 3 in *Cpe^fat/fat^* mice. *, p<0.05 using Student's two-tailed t-test.

To explore whether proSAAS and proNPY mRNA are co-regulated upon food deprivation, mice were fasted for 48 hours, mRNA was extracted from the hypothalamic subregions, and qRTPCR was performed. ProSAAS mRNA was not greatly affected by food deprivation in any hypothalamic region analyzed of WT mice (Figure 6A), whereas proNPY mRNA was significantly elevated, by approximately 150%, in the MBH of the food-deprived WT mice (Figure 6B). The increase in proNPY mRNA in the MBH is consistent with a previous report [28]. Because proSAAS-derived peptides were previously found to be elevated by food deprivation in *Cpe^fat/fat^* mice, we examined if proSAAS mRNA levels were affected by fasting in the mutant animals. As found for the WT mice, proSAAS mRNA levels were not greatly altered by food deprivation in the MBH or other hypothalamic regions in the *Cpe^fat/fat^* mice (Figure 6C). Levels of proNPY mRNA increased significantly in the MBH of the fasted *Cpe^fat/fat^* mice, but were not altered in other subhypothalamic brain regions (Figure 6D).

### Peptidomic analysis of proSAAS-derived peptide levels in fed and fasted wild-type mice

The previous study that found that food deprivation led to elevated levels of proSAAS-derived peptides was performed in *Cpe^fat/fat^* mice and examined peptide processing intermediates, not the mature forms of the peptides [21]. To test if food deprivation affected levels of the mature forms of the peptides in wild-type mice, relative levels of proSAAS-derived peptides were quantified using a mass spectrometry-based quantitative peptidomics technique. Peptide levels from the hypothalami of wild-type mice fed *ad libitum* were compared to levels in mice fasted for either 24 or 48 hours. Several peptides derived from proSAAS were detected in this analysis, including big LEN, little SAAS, PEN, and several smaller fragments of little SAAS and PEN (Table 1). None of these peptides showed a significant change between fed animals and either of the food-deprived groups (Table 1).

**Table 1:** Relative levels of proSAAS-derived peptides in fed and fasted mice.

Peptide name	Sequence	Theor. Mass	n	Cont A/B =	1 d fast/Cont	2 d fast/Cont
				Avg ± SD	Avg ± SD	Avg ± SD
Big LEN	LENPSPQAPARRLLPP	1754.98	4	0.98±0.06	0.97±0.13	1.06±0.06
Little SAAS	SLSAASAPLVETSTPLRL	1812.01	4	1.08±0.15	1.07±0.28	1.07±0.16
Little SAAS 1–16	SLSAASAPLVETSTPL	1542.81	4	1.05±0.13	0.96±0.15	1.06±0.20
Little SAAS 5–18	ASAPLVETSTPLRL	1453.81	2	1.12±0.01	1.09±0.09	1.03±0.21
PEN	SVDQDLGPEVPPENVLGALLRV	2316.23	4	0.95±0.12	0.96±0.15	1.02±0.26
PEN-18	(SVDQDLGPEVPPENVLGA)	1834.90	4	0.90±0.10	0.98±0.24	1.02±0.14
PEN-19	(SVDQDLGPEVPPENVLGAL)	1947.98	4	1.07±0.19	0.96±0.13	1.00±0.21
PEN-20	SVDQDLGPEVPPENVLGALL	2061.06	4	0.93±0.09	0.95±0.21	1.05±0.24

**Abbreviations:** Theor. Mass, theoretical monoisotopic mass of the peptide without isotopic tags or protons; n, number of runs in which the peptide was detected; Cont A/B, the relative level of peptide in two distinct groups of mice fed *ad libitum*; 1 d fast/Cont, the relative level of peptide in mice fasted for 1 day, relative to the level in control group B; 2 d fast/Cont, the relative level of peptide in mice fasted for 2 days, relative to control group B; Avg ± SD, average ratio of the 2–4 replicates with error range equal to the standard deviation. Most peptide sequences listed in column 2 were identified using MS/MS sequence information, in addition to several other criteria (mass within 0.005% of a known peptide, and the correct number of isotopic tags incorporated and charge state based on amino acid composition; see Materials and Methods) . Peptides surrounded by parentheses are tentatively identified by matches to known peptides.

### Injection of proSAAS-derived peptides in mouse hypothalamus; effects on acute food intake

**Figure 7:**
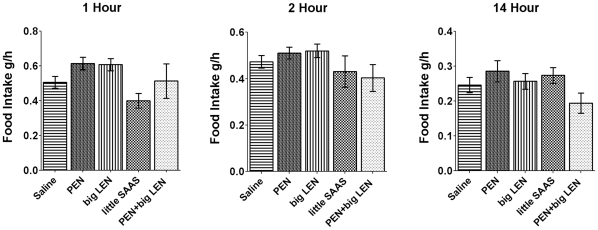
Comparison of food intake in mice injected with proSAAS-derived peptides. Mice do not show a significant peptide-induced change in food intake measured 1 hour, 2 hours, or 14 hours after the injection of either saline or the proSAAS-derived peptides: PEN, big LEN, little SAAS, or a combination of PEN and big LEN. Altogether 18 mice were cannulated and after recovery from surgery, injected with either 10 µg of peptide in saline or with saline alone. After 3–7 days of recovery, the mice were re-tested with the saline and peptide groups switched so that over the course of the study, each mouse received at least one control saline injection and injection of at least one of the different peptides. Error bars show standard error of the mean for saline (n = 37); PEN (n = 15); big LEN (n = 15); little SAAS (n = 8); and the combination of PEN and big LEN (n = 16).

The colocalization of proSAAS peptides with the orexigenic peptide NPY, as well as the phenotypes of proSAAS transgenic and proSAAS knockout mice [18], [19], indicate a possible orexigenic role for proSAAS-derived peptides. In order to test the effects of these peptides on food intake, we injected mice in the third ventricle with 10 µg of either PEN, big LEN, little SAAS, or a combination of PEN and big LEN peptides. Food intake was measured at 1, 2 and 14 hours post injection and compared to food intake in saline-injected control mice. None of the proSAAS-derived peptides showed a significant effect on food intake at any of the recorded time points (Figure 7).

### Injection of antibodies against proSAAS-derived peptides in mouse hypothalamus; effects on food intake

**Figure 8:**
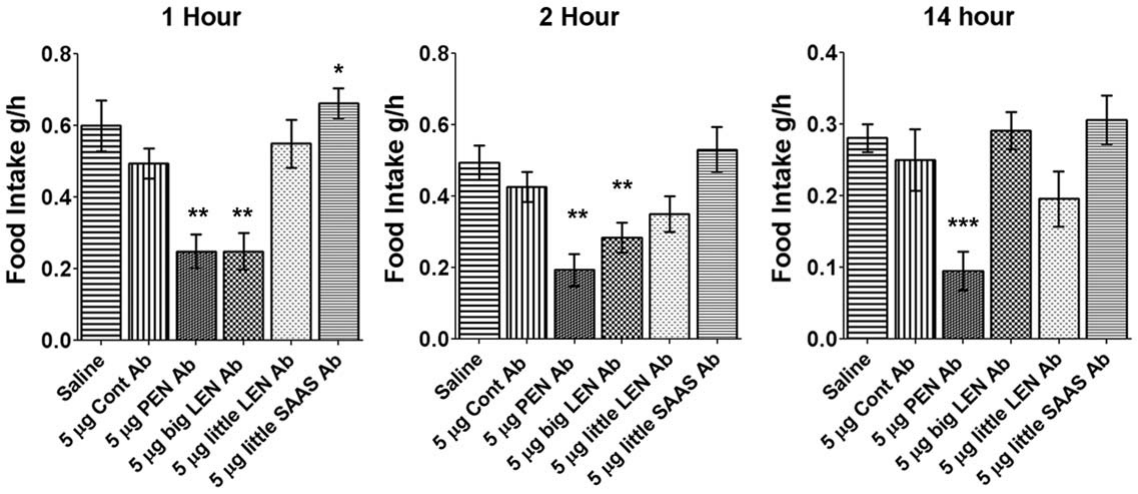
Comparison of food intake in mice injected with purified antibodies directed against proSAAS-derived peptides. Altogether, 18 mice were cannulated and in each experiment the mice were injected with either saline, control antibody (Cont Ab), or one of the antibodies directed against proSAAS-derived peptides, and food intake was measured 1 hour, 2 hours, and 14 hours after the injection. The antibody to PEN significantly reduced food intake at all time points examined. The antibody to big LEN showed a significant decrease in food intake after 1 and 2 hours, but not after 14 hours. In contrast, the antibody to little LEN did not significantly affect food intake at any time point examined, and the antibody to little SAAS showed a slight increase after 1 hour, but not at the other time points. Error bars show the standard error of the mean; saline (n = 21); control Ab (n = 19); PEN Ab (n = 8); big LEN Ab (n = 20); little LEN Ab (n = 10); little SAAS Ab (n = 8). *, p<0.05, **, p<0.01, ***, p<0.001 relative to control Ab, using Student's two-tailed t-test.

Because the proSAAS-derived peptides may not be stable upon icv injection into mouse brain, we tested whether icv injections of purified antibodies directed against the proSAAS peptides PEN, big LEN, little LEN and little SAAS affected food intake. At the one-hour time point, there was a significant decrease in food intake for mice injected with antibodies directed against PEN and big LEN (Figure 8). These mice ate approximately 50% as much as mice injected with saline or with a control antibody raised against a region of a *Drosophila* protein which has no counterpart in mammals. In contrast to the results with the antibodies to PEN and big LEN, there was no effect on food intake in mice injected with an antibody to little LEN. Mice injected with the antibody to little SAAS showed a small but significant increase in food intake relative to control antibody, although this increase was not significant compared to the saline injection (Figure 8). At the two hour time point, both PEN and big LEN antibodies continued to exert their anorexigenic effects, as these mice ate significantly less than mice injected with control antibody or saline (Figure 8). In contrast, mice injected with the little SAAS antibody showed no significant difference compared to the control group at the 2 hour time point. The effect of the PEN antibody on food intake was still observed after 14 hours, whereas mice injected with the antibody to big LEN returned to normal eating levels relative to controls by this time point.

### Electrophysiological analyses

**Figure 9:**
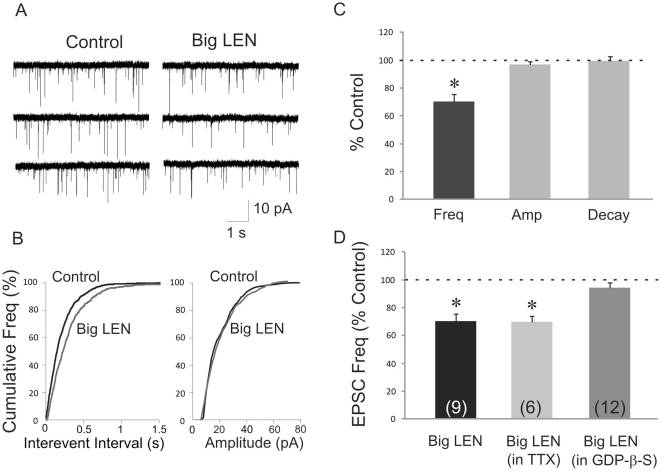
Big LEN suppresses excitatory synaptic input to PVN parvocellular neurons via the release of a retrograde messenger. A. Whole-cell patch clamp recording of spontaneous synaptic currents in a parvocellular neuroendocrine cell in the PVN in the absence (Control) and presence of big LEN (Big LEN). Big LEN (1 µM) caused a decrease in the frequency of negative synaptic currents, which are EPSCs mediated by glutamate release. B. Cumulative frequency plots of EPSC interevent intervals and EPSC amplitudes taken from the recording shown in A. Big LEN caused a shift toward longer interevent intervals, indicative of a decrease in EPSC frequency, but had no effect on the cumulative amplitude distribution. C. Mean frequency, amplitude and decay time of EPSCs in big LEN as a percentage of control measures. Big LEN (1 µM) decreased the frequency of EPSCs, but had no effect on EPSC amplitude or decay time (n = 9), suggesting a presynaptic suppression of glutamate release, but no effect on postsynaptic glutamate sensitivity. D. The effect of big LEN was maintained in TTX, indicating that the effect was action potential independent, but was blocked by an inhibitor of G protein activity (GDP-β-S) applied via the patch pipette into the postsynaptic cell, suggesting that the effect is mediated by the activation of a postsynaptic G protein-coupled receptor and release of a retrograde messenger.

Together with previous studies, the results described above suggest that proSAAS peptides PEN and big LEN function as neuropeptides. To explore this in more detail, we focused on big LEN using whole-cell patch clamp recordings in neurons located in the medial parvocellular region of the PVN in acutely prepared hypothalamic slices from rats. Putative neurosecretory parvocellular neurons were identified on the basis of their visualized position within the PVN and on electrophysiological criteria [29], [30]. The peptide big LEN (1 µM) applied in the bath perfusion had no effect on the membrane holding current required to clamp the cell at −60 mV (baseline: −21.1±6.1 pA; big LEN 25.6±8.2 pA; *p* = 0.51; n = 9) or on the mean input resistance (baseline: 723.3±76.6 MΩ; big LEN 708.2±87.8 MΩ; *p* = 0.77; n = 9). These results suggest that big LEN has no direct postsynaptic effect on the PVN parvocellular neuron passive properties. Big LEN (1 µM) application did, however, have an inhibitory effect on excitatory synaptic activity in PVN parvocellular neurons, causing a significant decrease in the frequency of glutamate-mediated spontaneous excitatory postsynaptic currents (EPSCs) (to 70.5±4.9% of baseline; *p*<0.05; n = 9 cells), but not on the EPSC amplitude (96.7±2.4% of baseline; *p* = 0.88) or decay time (99.4±3.2% of baseline; *p* = 0.55) (Figure 9). The big LEN-induced suppression of excitatory synaptic inputs to PVN parvocellular neurons had a rapid onset (3–5 min) and was reversible after 20–30 min of washout of the peptide. Blocking spike-dependent neurotransmitter release did not inhibit the effect of big LEN on glutamatergic inputs, as big LEN induced a decrease in spontaneous EPSC frequency in the presence of the voltage-gated sodium channel blocker tetrodotoxin (0.5 µM) to 69.8±4.2% of baseline (n = 6; *p*<0.05) (Figure 9D). This suggested that big LEN suppressed excitatory synaptic inputs to PVN parvocellular neurons by acting at presynaptic receptors on glutamatergic terminals.

We next investigated the molecular mechanisms of the big LEN effect in PVN parvocellular neurons by testing for a postsynaptic G protein dependence. The G protein blocker GDP-β-S (0.5–1 mM) was applied through the patch pipette to block postsynaptic G protein activity in the recorded cells. Blocking postsynaptic G protein activity with GDP-β-S inhibited the decrease in EPSC frequency induced by big LEN (94.5±3.3% of baseline; *p* = 0.07; n = 12). Since GDP-β-S is membrane impermeant and its actions are restricted to the postsynaptic cell, this indicated that the effect of big LEN on glutamate release was dependent on postsynaptic G protein activity in the parvocellular neurons, and implicates the involvement of a retrograde synaptic messenger that suppresses presynaptic glutamate release.

## Discussion

The major finding of the present study is that proSAAS-derived peptides have properties consistent with a function as neuropeptides involved in the regulation of feeding. One of the findings that support this role for proSAAS-derived peptides is their general colocalization with NPY. This conclusion was supported by immunofluorescence with antisera to several proSAAS-derived peptides and two different approaches to visualize NPY-positive neurons; an antibody to NPY peptide, and the GFP reporter under the control of the NPY gene promoter. There was no observed colocalization of proSAAS-derived peptides with α-MSH, an important anorexigenic peptide. This supports an orexigenic role for the proSAAS-derived peptides, which fits with previous studies on transgenic mice overexpressing proSAAS, which were slightly overweight, and proSAAS knock-out mice, which showed reduced body weight when male mice were examined [18], [19]. NPY is a major orexigenic neuropeptide, which transits the regulated secretory pathway and is secreted in response to signals from the body (ghrelin, amylin, vagus nerve signals, etc). NPY impinges upon receptors found on second order neurons in the PVN, the dorsomedial hypothalamus (DMH), and the LH [31]. ProSAAS-derived peptides which colocalize with NPY in many neurons should therefore be co-released onto the NPY-target neurons. While the NPY, big LEN and PEN peptides do not colocalize completely, this is consistent with their processing. NPY is processed from its precursor at a Lys-Arg site, while most of the processing of the proSAAS peptides occurs at either Arg-Arg or single Arg sites [11], [32]. The prohormone convertases show overlapping but distinct substrate specificities, which would explain why certain neurons with specific cohorts of endopeptidases would show overlapping localization of NPY and proSAAS-derived peptides, while others may process only one set of peptides or the other [33]. The punctate vesicular pattern of PEN and big LEN supports the hypothesis that these peptides are processed and released from the regulated secretory system, consistent with previous results finding that proSAAS expressed in a mouse corticotrophic cell line was secreted via the regulated pathway [11] and that proSAAS-derived peptides were released from cultured hypothalamic slices [34].

Although the distribution of proSAAS mRNA was found to be highest in the arcuate nucleus when analyzed by *in situ* hybridization in a previous study from our group [11] and in the Allen Mouse Brain Atlas (Figure S1), high levels of proSAAS mRNA expression are found in other brain regions such as the PVN, LH, suprachiasmatic nucleus, bed nucleus of the stria terminalis, medial preoptic area, paraventricular thalamus, piriform cortex, and multiple areas of the amygdala and hippocampus. Quantitative real-time PCR of proSAAS mRNA confirmed that MBH had the highest levels, although levels in PVN and LH were only slightly lower than MBH. Analysis of the same RNA samples showed very high levels of proNPY mRNA in the MBH and much lower levels in the other regions measured, which fits well with the Allen Mouse Brain Atlas (Figure 1), the observed distribution using the NPY antibody (Figure 2), the expression of GFP under the NPY gene promoter (Figure 3) and previous studies [35]. While the hypothalamic distribution of proSAAS mRNA is consistent with an orexigenic role for the proSAAS-derived peptides, the broader distribution in non-orexigenic neurons supports a potential role for proSAAS-derived peptides in other behaviors. For example, the proSAAS KO mice were found to show behavior consistent with elevated anxiety when placed in an open field [19].

Previous studies have shown an increase in proSAAS-derived peptide levels in the whole hypothalamus of the *Cpe^fat/fat^* mouse after a 48 hour fast using the quantitative peptidomics technique [21], [36]. These previous studies however did not find up-regulation of proSAAS mRNA in the whole hypothalamus as assessed by Northern blot. Therefore, one aim of the present study was to characterize proNPY and proSAAS mRNA regulation in specific subhypothalamic brain regions of the mouse hypothalamus upon fasting for 48 hours. ProNPY mRNA levels showed significant up-regulation with fasting specifically in the MBH, which concurs with previous studies examining the regulation of proNPY mRNA in the arcuate nucleus of rats and mice following a two day fast [28], [37], [38], [39]. ProSAAS mRNA was not significantly upregulated by fasting in any of the hypothalamic regions analyzed, and was even slightly decreased in the MBH. The increase in proSAAS-derived peptide levels observed in *Cpe^fat/fat^* mouse hypothalamus after a 48-hour fast in the previous experiments is therefore likely to be regulated at the translational or post-translational level. Furthermore, while a 2-day fast was previously found to increase several proSAAS-derived peptides in *Cpe^fat/fat^* mouse hypothalamus, neither a 1- nor a 2-day fast was found in the present study to alter the major proSAAS-derived peptides detected in wild-type mouse hypothalamus. This is consistent with the absence of a change in proSAAS mRNA levels upon fasting in most hypothalamic regions of wild-type animals, and suggests a complex regulatory difference between wild-type and *Cpe^fat/fat^* animals. ProSAAS mRNA levels in the MBH of the *Cpe^fat/fat^* mice were significantly lower than in the wild-type mice. There was also a decrease in the level of proNPY mRNA in the MBH of *Cpe^fat/fat^* mice relative to wild-type mice, though it did not reach statistical significance. It is possible that these changes are secondary to the animals' obesity, due perhaps to effects of leptin or other peripheral signals on proNPY and proSAAS expression.

It has been previously shown that intracerebroventricular administration of NPY and some other neuropeptides can acutely affect food intake in rodents [23]. Intracerebroventricular injection of several proSAAS-derived peptides was performed to assess their effect on acute feeding. None of these peptides was able to elicit an acute effect on feeding in wild-type mice. This does not necessarily mean, however, that these peptides are not involved in feeding or bodyweight regulation because the injected peptides may not be stable; there are a number of extracellular peptidases that rapidly degrade many different peptides. In previous peptidomic studies, many small proSAAS-derived peptides have been detected which result from cleavages at nonbasic sites [33], [40]. These smaller peptides are presumably generated by the action of extracellular proteases, which would mean that proSAAS-derived peptides are substrates of these enzymes. Introcerebroventricluar injection of angiotensin II was preformed to verify proper cannulation, and as all mice responded by increased drinking behavior, the injection coordinates and technique are unlikely to be the explanation for the lack of response to proSAAS derived peptides. Thus, a second approach was used; blocking peptide function by the injection of antibodies. Previous studies have used antibodies to block the biological effect of neuropeptides [24]. Injection of the purified big LEN and PEN antibodies showed acute effects on food intake relative to a control antibody and to saline. The decrease in food intake observed with these antibodies is consistent with the orexigenic role proposed for the proSAAS peptides, and suggested by their localization and the phenotypes of knockout and transgenic animals. In contrast, the other antibodies tested did not show a consistent change relative to either saline or the control antibody. Thus, while big LEN appears to affect feeding, little LEN does not. These two peptides differ in the C-terminal region, with little LEN being 6 amino acids shorter than big LEN. There are many examples where the extent of processing of neuropeptide precursors influences the resulting biological activity [41].

A number of different neuropeptides are known to modulate neuronal activity through various mechanisms. We tested big LEN on cells in a brain region influenced by NPY. We postulated that, because big LEN is colocalized with NPY in many neurons within the arcuate nucleus, it should also be co-released with NPY and potentially modulate a neuronal target of NPY projections, the parvocellular neuroendocrine cells in the PVN [42]. The finding that big LEN suppressed glutamate release onto PVN parvocellular neurons is further evidence that this peptide is functional as a neuropeptide. Furthermore, the postsynaptic G protein dependence and the TTX insensitivity of the effect suggest that big LEN inhibits glutamate release via postsynaptic G protein activation, suggesting a postsynaptic site of peptide action and involvement of a retrograde messenger. Taken together, our data suggest that the peptide big LEN suppresses excitatory synaptic input to PVN parvocellular neurons by activating a G protein-coupled receptor on the PVN parvocellular neurons and stimulating the release of a retrograde messenger, which acts on presynaptic terminals to suppress glutamate release. Thus, big LEN has the cellular distribution and functional properties to be considered a neuropeptide involved in the regulation of food intake.

## Materials and Methods

### Reagents

Unless indicated, reagents were obtained from Sigma-Aldrich (St.Louis, MO, USA). Big LEN and PEN antisera used in these experiments were previously generated in rabbit [43]. The cross reactivity of these antisera with other proSAAS-derived peptides has been previously reported [44]. A chicken antiserum to the peptide PEN-LEN was also previously generated and characterized [14]. Commercially available anti-NPY (Phoenix Pharmaceuticals, Burlingame, CA, USA) and anti-α-MSH (Millipore, Billerica, MA, USA) antisera were used. Little LEN antiserum was the generous gift of Dr. Iris Lindberg (Dept. Anatomy and Neurobiology, University of Maryland-Baltimore, MD, USA).

### Animals

Male C57BL/6J mice (Jackson Laboratory, Bar Harbor, ME) were maintained in individual cages on a 12-h light/dark cycle. NPY-GFP mice (B6.FVB-Tg(Npy-hrGFP)1Lowl/J) mice were the gift of Dr. Clemence Blouet and Dr. Gary Schwartz (Albert Einstein College of Medicine), and were originally obtained from Jackson Laboratory (Bar Harbor, ME). Mice were 8–10 weeks of age at the start of all experiments. All efforts were made to minimize the suffering and the number of animals used. The experiments were approved by the Institutional Animal Care and Use Committee and the Institute for Animal Studies of Albert Einstein College of Medicine (protocol number 20090305).

### Tissue preparation for immunohistochemistry

Mice were anaesthetized using diethyl ether and perfused transcardially through the ascending aorta with 4% buffered paraformaldehyde. Brains were postfixed overnight in 4% buffered paraformaldehyde at 4°C, subsequently dehydrated in 30% sucrose in phosphate buffered saline overnight, embedded in Tissue-Tek Optimal Cutting Temperature compound (Sakura Finetek, Torrance, CA, USA) and frozen in isopentane (Fisher Scientific Hampton, NH, USA) at −50°C and stored at −70°C until cutting. Coronal sections of 14 µm were cut on a sliding microtome in the region of the arcuate nucleus of the hypothalamus and thaw mounted onto Superfrost Plus microscope slides (Fisher Scientific, Hampton, NH, USA).

### Immunohistochemistry

Sections on slides were washed with PBS, then blocked for 2 hours at room temperature in 5% bovine serum albumin (BSA) in PBS containing 0.5% Triton X-100. Slides were then incubated for 24 hours at 4°C with either chicken anti-PEN-LEN (1∶20,000), rabbit anti-big LEN #85 (1∶10,000), rabbit anti-PEN #142 (1∶10,000), rabbit anti-NPY (1∶200, Phoenix Pharmaceuticals, Burlingame, CA, USA) or sheep anti-α-MSH (1∶5000, Millipore, Billerica, MA, USA) in 5% BSA in PBS with 0.5% Triton X-100. After washing with PBS containing 0.2% Tween-20, secondary Cy3- and Cy2-conjugated antibodies (Jackson Immunoresearch, West Grobe, PA, USA) were applied. After overnight incubation at 4°C, the sections were washed and mounted in antifade reagent containing 4,6- diamidino-2-phenylindole (DAPI; Invitrogen, Carlsbad, CA, USA). To control for non-specific staining, preimmune serum from each of the animals used for the proSAAS-derived peptide antisera were applied to adjacent sections at the same dilutions as the antisera (Figure S2). Additional controls were performed by preincubating the antisera with the appropriate peptide prior to incubation with the tissue (Figure S2).

### RNA extraction

Male mice between 9 and 10 weeks old were fasted or fed *ad libitum* for 48 hours prior to sacrifice by cervical dislocation and brains were excised. Three mice were used for each of the fasted and fed conditions. Brains were cut into 1.5 mm coronal sections. The mediobasal hypothalamus, the bilateral lateral hypothalamus, and the paraventricular nucleus were dissected from the section containing the arcuate nucleus, bregma −1.3 mm to −2.8 [45] Brain regions were immediately frozen and stored at −70°C until extraction. RNA extraction was performed using 500 µL Qiazol (Qiagen) reagent per brain region. Tissues were sonicated for ten pulses (50% duty cycle, level 3, one second per pulse). Qiagen Lipid RNeasy Mini RNA extraction kit (Qiagen) was used according to manufacturer's directions, and RNA was eluted in 20 µL of RNAse free water. cDNA was then generated using the Superscript III First Strand Synthesis System for RTPCR (Invitrogen). Random hexamer primers were used with 400 ng extracted RNA per brain region.

### Quantitative real-time PCR (qRTPCR)

qRTPCR was performed using the following primer sets (Invitrogen, Carlsbad, CA, USA): proNPY (Forward 5′-GTTTGGGCATTCTGGCTGAGGG, Reverse 5′-TGTCTCAGGGCTGGATCTCTTGC), proSAAS (Forward 5′-ATTTTGGTGCTGCTGCTCTT, Reverse 5′-GGAGTGCTCGTCTCAACCA) and glyceraldehyde 3-phosphate dehydrogenase (GAPDH: Forward 5′-AGATTGTTGCCATCAACGAC, Reverse 5′-TTGACTGTGCCGTTGAATTT). All primer sets were intron-spanning and were verified by cDNA transcript length by amplification using SuperScript III One-Step RT-PCR System with Platinum Taq DNA polymerase (Invitrogen, Carlsbad, CA, USA) and gel electrophoresis. cDNA generated by first strand synthesis was used to quantify RNA levels by qRTPCR using Power SYBR Green PCR master mix (Applied Biosystems, Warrington, UK) in 384 well plates on an ABI 7900HT Fast Real-time PCR system (Applied Biosystems, Warrington, UK) using a SYBR 3-step protocol at the Albert Einstein College of Medicine genomics core facility. Data analysis was performed using SDS software (Version 2.1, Applied Biosystems, Warrington, UK). Fold change in RNA was calculated from qRTPCR results using the ΔΔCT method. To calculate the levels of proSAAS and proNPY mRNA among the brain regions analyzed, the levels in the PVN and the LH were compared to the levels in the MBH, which was set as one. To calculate the difference in proSAAS and proNPY mRNA levels between wild-type and *Cpe^fat/fat^* mice, the level of mRNA in each wild-type brain region was set as one, and levels in the *Cpe^fat/fat^* mice were compared to those levels. In the comparison of the fed and fasted wild-type mice, the level of each mRNA in the fed mice brain regions was set at one, and the mRNA levels in the fasted mice were compared to those levels. Significance of changes was calculated using the two-tailed Student's t-test.

### Peptidomic analysis of hypothalamic peptides under variously fed and fasted conditions

Thirty two male mice were split into four groups of eight mice each. Each group of mice was kept on a separate feeding schedule. These feeding schedules consisted of a 24 hour fast, a 48 hour fast, and two groups that were fed *ad libitum* during the experimental period. All mice were 7–8 weeks old at the time of the experiments. Mice were sacrificed and tissues were collected as previously described [33]. Tissue was frozen in dry ice and stored at −70°C until analysis. Tissue was extracted and labeled as as previously described [46], [47].

To detect and quantify the labeled peptides from fasted and fed mouse hypothalami, liquid chromatography and tandem mass spectrometery (LC/MS/MS) was performed on a Waters Q-TOF - Ultima Mass Spectrometer (Micromass, Manchester, UK) as previously described [48]. The most intense ion in each MS survey scan was fragmented to generate MS/MS spectra. Peptides were identified as previously described [33], [47], [49]. To reduce the number of false-positives, all Mascot hits were manually interpreted as previously described (Berti et al. 2009;Morano et al. 2008;Zhang et al. 2008). Quantitation was performed as previously described [20], [47]. In brief, the peak intensity for the monoisotopic peak and the peak containing one ^13^C atom were averaged. In spectra with overlapping peaks in which the signals with multiple ^13^C atoms extended into the range of the next TMAB tag, the isotopic distribution of the peptide was calculated and the contribution from the ^13^C-containing peaks subtracted, as described [47]. Each LC/MS run contained tissue extract from a group of 24 hour-fasted mice, a group of 48 hour-fasted mice, and two groups of mice fed *ab libitum*. The relative peak intensity for each of these groups was determined for each of the three replicates, and the ratios of these replicates were averaged.

### Antibody purification

Protein A agarose columns (Invitrogen, Carlsbad, CA, USA) were used for purification of antibodies prior to icv injection according to manufacturers' instructions. The IgG concentration of each elute was determined using a spectrophotometer.

### Cannulation

Mice were anaesthetized using isoflurane and fixed by ear bars in a stereotactic apparatus. The top of the mouse head was shaved and cleaned using sterile cotton swabs, betadine solution and isopropyl alcohol solution. Once dry, a small section of the scalp was removed with scissors. The exposed skull was then cleaned with sterile saline followed by 30% hydrogen peroxide solution. After drilling a hole in the skull, the guide cannula (Plastics One, Roanoke VA) was inserted at bregma −1.82 mm to a depth of bregma −5 mm. The guide cannula was held in place using metals screws and dental cement, and occluded to prevent clogging using a 5.5 mm obturator. Mice were allowed to heal for 7–10 days before injections were performed.

### Peptide and antibody injections

Mice were initially injected with 50 ng angiotensin II in 2 µL sterile saline and observed for drinking response to verify cannulation into the third ventricle. All mice showed a marked stimulation of water consumption following the angiotensin II injection.

In subsequent studies, mice were fasted for 8 hours prior to lights out. For injection of antibodies to proSAAS-derived peptides or a control antibody generated against a *Drosophila* antigen, 5 µg of purified antibody in 2 µl of injectate was infused by hand at a rate of 0.5 µl/minute, using a 5 µl Hamilton syringe (Hamilton Company, Reno NV) attached by PE20 tubing, 0.38 mm inside diameter (Plastics 1, Roanoke VA) to an injector cannula (Plastics 1) which protruded 0.5 mm below the bottom of the guide cannula. For injection of proSAAS peptides, 10 µg of peptide in 2 µl sterile saline (or saline alone as a control) was infused at 0.5 µl/minute. After injection, the obturator was replaced, the mouse was returned to his home cage, and food was provided. In a typical experiment, 6–8 mice were injected with a single peptide or antibody to a proSAAS-derived peptide, and an equal number received the control saline or control antibody injection. All mice received injections before lights out, timed so that the food measurements were performed during the dark cycle. The quantity of food remaining was measured one hour, two hours, and 14 hours post-injection. After 4–7 days of recovery, the experiment was repeated with groups switched so that all mice received both control and big LEN antibodies and at least one of the PEN, little LEN or little SAAS antibodies.

### Tissue preparation for patch-clamp electrophysiology

Male Wistar rats (4–6 weeks old, Harlan Laboratories, Indianapolis, IN) were used in these experiments according to a protocol approved by the Tulane University Institutional Animal Care and Use Committee. Rats were decapitated under ketamine-xylazine anesthesia and the brain was quickly removed from the skull. Two to three 300- µm coronal hypothalamic slices containing the bilateral paraventricular nuclei were sectioned on a vibratome (Vibratome, St. Louis, MO) in ice-cold artificial cerebral spinal fluid (aCSF), in which NaCl was replaced by an equimolar concentration of sucrose to improve neuronal viability. The standard aCSF contained 140 mM NaCl, 3 mM KCl, 1.3 mM MgSO_4_, 1.4 mM NaH_2_PO_4_, 2.4 mM CaCl_2_, 11 mM glucose, and 5 mM HEPES, bubbled with 100% O_2_. The slices were bisected at the third ventricle, and each half slice was submerged in a holding chamber in oxygenated aCSF at room temperature, where it was allowed to equilibrate for 1.5–2 h before being transferred to the recording chamber.

### Patch-clamp recordings

After transfer to the recording chamber, slices were allowed to equilibrate for >15 min and maintained at a temperature of 32–34°C throughout recordings. Paraventricular nucleus medial parvocellular neurons were targeted by direct visualization under infrared illumination and differential interference contrast optics. Patch electrodes were pulled to a tip resistance of 3–4 MΩ on a Flaming-Brown horizontal pipette puller (P-97, Sutter Instruments, Novato, CA). The internal recording solution contained 120 mM K-gluconate, 10 mM KCl, 1 mM NaCl, 1 mM MgCl_2_, 1 mM CaCl_2_, 10 mM EGTA, 2 mM Mg-ATP, 0.3 mM Na-GTP, and 10 mM HEPES. Cells with unstable input resistance or series resistance were discarded. All recordings were performed in voltage clamp mode using a Multiclamp 700A amplifier and pCLAMP 9 software (Molecular Devices, Sunnyvale, CA). Data were low-pass filtered at 2 kHz, digitized at 5 kHz and recorded for off-line analysis. In a subset of neurons, tetrodotoxin (TTX, 1 µM) was added to the aCSF to block spike-mediated transmitter release. Postsynaptic currents were analyzed for changes in frequency, amplitude and decay time (defined as the time from peak to 63% decay) using the Minianalysis 6.0 program (Synaptosoft Inc., Decatur, GA).

### Electrophysiological data analysis

All data are expressed as means ± standard errors. Statistical comparisons of electrophysiological data were performed using the Student's paired t-test for within-group cell comparisons, the Student's unpaired t-test for between-group comparisons. P values of <0.05 were considered significant for all comparisons.

## Supporting Information

Figure S1 **ProSAAS, proSAAS-derived peptides, and the distribution of proSAAS mRNA in mouse brain.**
(PDF)

Figure S2 **LEN antibodies are specific for the peptide big LEN.**
(PDF)
