# Supplementary material for: ProSAAS-Derived Peptides are Colocalized with Neuropeptide Y and Function as Neuropeptides in the Regulation of Food Intake
Source: PLoS One. 2011 Dec 2;6(12):e28152. doi: 10.1371/journal.pone.0028152 (PMC3229528; doi:10.1371/journal.pone.0028152)

Figure S1. ProSAAS, proSAAS-derived peptides, and the distribution of proSAAS mRNA in mouse brain. A: Schematic diagram showing the major proSAAS-derived peptides previously detected in various brain regions and other relevant peptides. The relative size and position of these peptides within proSAAS are indicated. Major peptides include big SAAS (ARPVKEPRSLSAASAPLVETSTPLRL), little SAAS (SLSAASAPLVETSTPLRL), KEP (ARPVKEPR), GAV (AVPRGEAAGAVQELARALAHLLEAERQE), PEN (SVDQDLGPEVPPENVLGALLRV), big LEN (LENPSPQAPARRLLPP), and little LEN (LENPSPQAPA). Smaller forms of some of these peptides have been detected in mouse brain (for example, see Table 1). In addition to these peptides, PEN/LEN (SVDQDLGPEVPPENV-LGALLRVKRLENPSPQAPARRLLPP) is indicated; this has not been detected by mass spectrometry in mouse brain, but is predicted to be a peptide-processing intermediate of proSAAS. Synthetic PEN/LEN, but not the mature forms PEN and LEN, is a potent inhibitor of PC1/3. All sequences indicated above are mouse (*Mus Musculus*). B-D: Images were downloaded from the Allen Mouse Brain Atlas [Internet]. Seattle (WA): Allen Institute for Brain Science. ©2009. Available from: http://mouse.brain-map.org (Lein ES, Hawrylycz MJ, Ao N, Ayres M, Bensinger A, et al. (2007) Genome-wide atlas of gene expression in the adult mouse brain. Nature 445: 168-176). B: ProSAAS mRNA expression in a sagittal section of mouse brain. C: The gene expression filter was used with the image shown in panel B to provide relative quantitation of the levels of proSAAS mRNA. Inset: relative level scale bar. D: Anatomical map of the sagittal plane shown in panels B and C. For definitions of abbreviations, see the Allen Brain Atlas website.

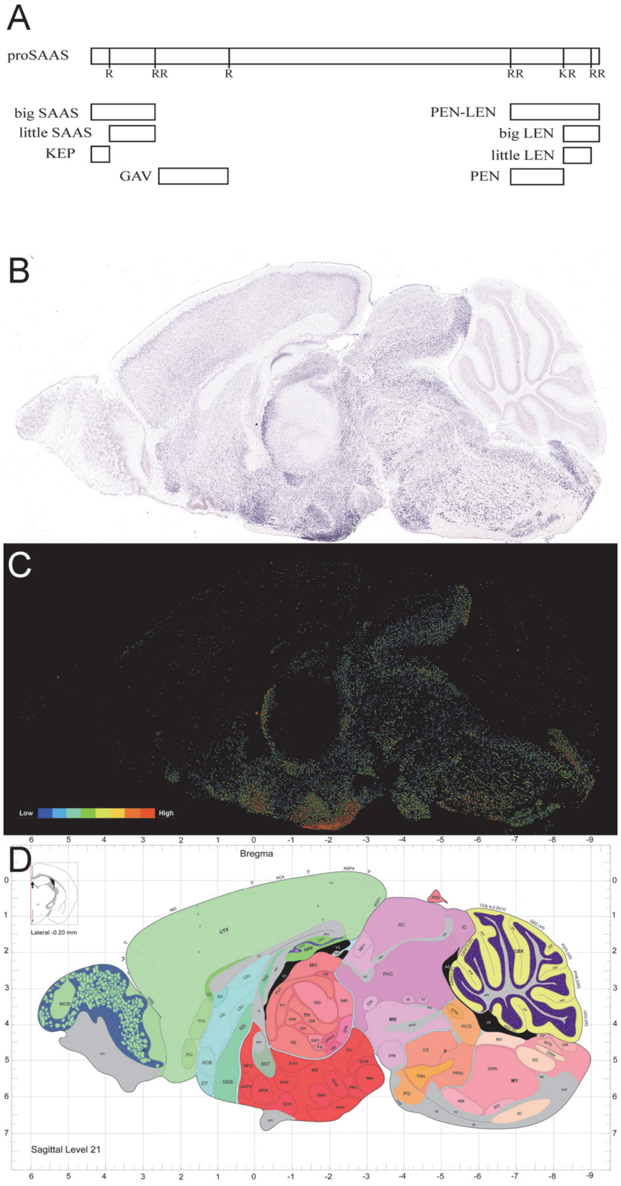

Supplement: Figure S1 — ProSAAS, proSAAS-derived peptides, and the distribution of proSAAS mRNA in mouse brain. (PDF) [file pone.0028152.s001.pdf]
