# Supplementary material for: ProSAAS-Derived Peptides are Colocalized with Neuropeptide Y and Function as Neuropeptides in the Regulation of Food Intake
Source: PLoS One. 2011 Dec 2;6(12):e28152. doi: 10.1371/journal.pone.0028152 (PMC3229528; doi:10.1371/journal.pone.0028152)

Figure S2: LEN antibodies are specific for the peptide big LEN. The upper panel depicts the area surrounding the third ventricle of the mouse hypothalamus. The left panel shows staining of mouse brain with the big LEN antibody under standard conditions, as used in Figures 2 and 3. The right panel shows results when the anti-LEN antiserum was preincubated with big LEN peptide prior to staining. Note the decrease in signal with the blocked antiserum. Scale bar for the top two panels = 50 µm. The middle row of panels shows the same panels as in the top row, but analyzed with higher magnification. The bottom panels show the lack of staining by preimmune sera from the chicken used to raise antiserum to PEN/LEN (left panel) and the rabbit used to raise antiserum to LEN (right panel). Scale bar for the lower 4 panels, indicated in the lower right panel = 10 µm.

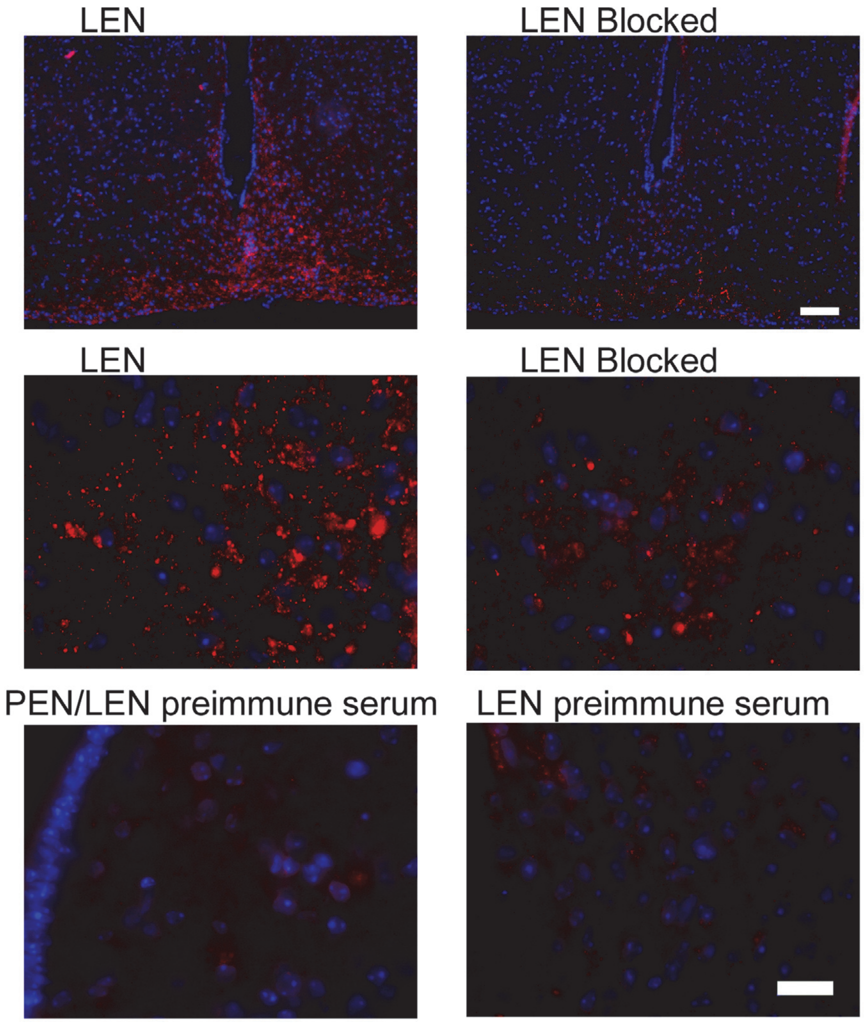

Supplement: Figure S2 — LEN antibodies are specific for the peptide big LEN. (PDF) [file pone.0028152.s002.pdf]
